# The Relationship Between Body Mass Index and Different Regional Patterns of Lymph Node Involvement in Papillary Thyroid Cancers

**DOI:** 10.3389/fonc.2021.767245

**Published:** 2021-12-22

**Authors:** Changlin Li, Gianlorenzo Dionigi, Nan Liang, Haixia Guan, Hui Sun

**Affiliations:** ^1^ Division of Thyroid Surgery, China-Japan Union Hospital of Jilin University, Jilin Provincial Key Laboratory of Surgical Translational Medicine, Jilin Provincial Engineering Laboratory of Thyroid Disease Prevention and Control, Changchun City, China; ^2^ Division of Surgery, Istituto Auxologico Italiano IRCCS, Milan, Italy; ^3^ Department of Medical Biotechnology and Translational Medicine, University of Milan, Milan, Italy; ^4^ Department of Endocrinology, Guangdong Provincial People’s Hospital, Guangdong Academy of Medical Sciences, Guangzhou, China; ^5^ The Second School of Clinical Medicine, Southern Medical University, Guangzhou, China

**Keywords:** body mass index, lymph node, papillary thyroid cancer, metastasis, regional patterns

## Abstract

**Introduction:**

Lymph node (LN) metastasis is the first site of metastasis of papillary thyroid cancer (PTC). LN status influences clinical management and the prognosis of patients. We explored the relationship between patient obesity and regional patterns of LN involvement in PTC.

**Materials and Methods:**

This study retrospectively analyzed the data from 12,772 PTC patients. The rate of LN metastasis, number of LN metastasis, maximum diameter of positive LN, number of *dissected LN*, and LN ratios (LNR) were compared between normal-weight and obese patients. Statistical methods have been adjusted for the confounders in hypothesis testing.

**Results:**

Overweight and obesity were independent risk factor for metastatic LNs (OR_1_ = 1.125, 95% CI 1.042-1.214, *P_1_ = *0.003; OR_2_ = 1.554, 95% CI 1.339-1.802, *P_2_* <0.001). Obesity was an independent risk factor for the number of metastatic CLNs (OR=1.159, 95% CI 0.975-1.377, *P*=0.045), however not for number of metastatic lateral LNs (*P*=0.907). Furthermore, obesity was not an independent risk factor for number of CLNs when dissected more than five (*P*=0.653), still an independent risk factors for number of metastatic lateral LNs when more than six (OR=1.185, 95% CI 1.010-1.391, *P*=0.037). As for LNR, obesity was an independent risk factor for the central LNR when more than 0.12 (OR _adjusted 1_ = 1.099, 95% CI 1.011-1.194, *P_1_ = *0.027; OR _adjusted 2_ = 1.177, 95% CI 1.003-1.381, *P_2_ = *0.045), for the lateral LNR more than 0.05 (*P_2_ = *0.283).

**Conclusions:**

Obesity was associated with poor prognoses with PTC respecting LNs. Surgeons should be extreme caution when performing central neck dissection in obese patients.

## Introduction

The prevalence of obesity is approximately 40% worldwide, affecting more than 2 billion adults (1). Obesity has been identified as an independent risk factor for many cancers. Some studies have reported that nearly 40% of cancers may be attributed to obesity (2), and there is strong evidence to suggest that obesity is related to cancers of the esophagus, liver, pancreas, gallbladder, ovary, thyroid, kidneys, and plasma cells (3). Although obesity has been linked to an increased risk of diabetes and coronary artery disease, the impact of obesity on the incidence rates, risk factors, morbidity, and mortality of thyroid cancer requires further exploration (4). As thyroid cancer is the most common type of endocrine tumor, understanding how BMI impacts this disease has vital public health implications (5). Obesity also affects the diagnostic assessment of patients negatively. Deglise et al. found that obese women were less likely to have undergone ultrasound (OR=0.5) or MRI (OR= 0.3) and were at an increased risk of prolonged hospital stays (OR=4.7) in the clinic (6).

A significant association has been established between elevated BMIs and increased papillary thyroid carcinoma (PTC) incidence rates (7). In a previous study, obesity was identified as a risk factor for thyroid cancer, specifically when tumor sizes were larger than 1 cm with multifocality and extrathyroidal extensions (8–10). However, the connection between obesity and lymph node (LN) metastasis of thyroid cancer has not been thoroughly investigated. One of the most important prognostic factors in PTC is LN status (11). LN metastasis is the first station of extra-gland metastasis of thyroid cancer (11). Hence, LN metastasis is an excellent predictor of the prognosis of patients with thyroid cancer. LN status can influence important clinical decisions, such as therapeutic options (11). The American Thyroid Association (ATA) guidelines use the number of metastatic LNs and the maximum diameter of positive LNs as important indicators for predicting the risk of recurrence (11). Compared to patients with less than five metastatic LNs, the recurrence rates are much higher for patients with more than five metastatic LNs (19% vs. 8%) (12, 13). In terms of maximum positive LN diameter, recurrence rates are significantly higher in patients with the diameter of LNs higher than 3 cm (27% vs. 5%) (13).

In this study, we focused on the relationship between obesity and the patterns of LN involvement in PTC, with an emphasis on the number of metastatic LNs, the maximum diameter of positive LNs, the lymph node ratio (LNR), the number of dissected LNs, and the LN skip metastasis. 

## Materials and Methods

### Study Design

This study was a retrospective descriptive analysis of patients with operable PTC. The patients in this study were diagnosed with PTC at our Institute between June 2008 and December 2017. Patient data was gathered and stored in the Institutional database of the Division of Thyroid Surgery, China-Japan Union Hospital of Jilin University. The Institutional data collected have important impact locally on the epidemiological surveillance, evaluation of thyroidal disease, impact of the treatment on diseases progression and improvement on research quality.

**Figure 1:**
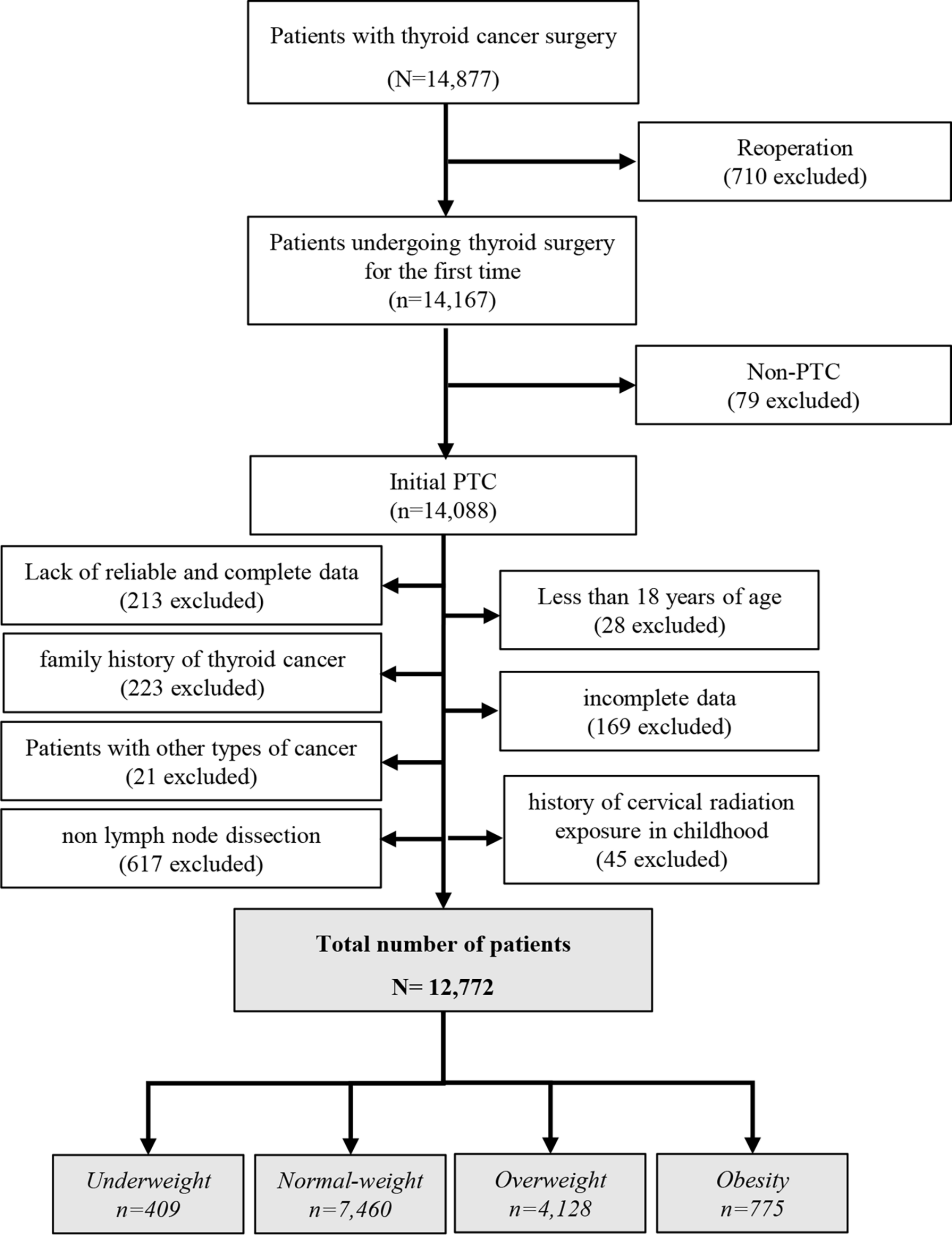
Flow chart of study inclusion and exclusion criteria.

All the patients eligible for this analysis received central neck node dissection. Patients were aged ≥18 years, pathologically-confirmed to have PTC, and PTC patients routinely underwent prophylactic central neck dissection (CND). The exclusion criteria was: non-PTC patients, other thyroid cancer subtypes, different types of cancer, family history of thyroid cancer; history of cervical radiation exposure in adolescence or childhood, incomplete data, no lymph node dissection, and patients requiring reoperations (**Figure 1**). Patients who do not fall into the criteria of this study will be excluded from the effectiveness data set, but all safety observation records will be kept in a safe center.

### Treatments

Oncological treatment, which ranged from surgery to radioactive iodine (RAI), was standardized for all patients and in accordance with the multidisciplinary tumor board consensus. The histological subtype was assessed according to the WHO classification. According to the Chinese guidelines for diagnosis and treatment of differentiated thyroid, all patients with thyroid cancer routinely underwent prophylactic CND (14). For those LNs suggested to be malignant by ultrasonography, fine needle aspiration cytology (FNAC) was used to confirm the diagnosis. Patients with cervical lymph node metastasis were confirmed by preoperative FNAC or intraoperative frozen pathological examinations. These patients underwent therapeutic cervical lymph node dissection. Prophylactic cervical lymph node dissection was not recommended. The upper bound of the range of CND is the lower hyoid bone, the lower bound is the superior sternum fossa, the outside is the common carotid artery, and the inside is the inside of the trachea. The lateral LN dissection minimum ranged from the IIa, III, IV, and Vb area, while other areas were treated according to the results of the FNAC.

### Definitions

Body-mass index. According to the WHO-BMI standard, underweight is <18.5 kg/m^2^, normal weight is 18.5‐24.9 kg/m^2^, overweight is 25‐29.9 kg/m^2^, and obese is ≥30 kg/m^2^ (15). Height and weight measurements used to calculate the BMI were retrieved from electronic registration databases. BMI was calculated as weight divided by height squared. Upon the first admission, the demographics and clinical data, including height and weight, were recorded.

*Pattern of metastatic LNs.* The pattern of metastatic LNs included the rate of metastatic LNs, the number of metastatic LNs, the number of dissected LNs, the lymph node ratio (LNR), the maximum diameter of positive LNs, and the rate of LN skip metastasis.

*Maximum diameter of positive LNs.* The maximal tumor diameter of the largest metastatic LN using the concept of micro-metastases in breast cancer (16).

*Lymph node ratio (LNR).* LNR was defined as the number of nodes involved by the tumor divided by the total number of resected lymph nodes during the surgical treatment.

*LN skip metastasis*. Defined as a lateral lymph node metastasis without central lymph node involvement.

### Pathological Examination

Specimens removed during the operation were submitted for histological analysis to determine the presence and size of metastatic LNs. For this study, an experienced pathologist reviewed the pathological slides and measured the properties of each LN.

### Outcomes and Covariates

We used BMI as an index and further correlated it with the presence and pattern of LN metastases. Patients were identified as LN positive if they had a pathologic LN status of pN1a or pN1b. Based on prior studies, we included important predictors of LN status in multivariable models (17). For example, information regarding the following thyroid tumor characteristics was obtained from medical records: age, weight, height, tumor size (mm), multifocality, extrathyroidal extension, rate of metastatic LNs (%), number of metastatic LNs, number of *dissected LN*, LNR (%), maximum diameter of positive LNs (mm), and LN skip metastasis (%).

### Sample Size Collection

Retrospective analysis of sample size (i.e., margin of error and level of confidence calculation) was carried out through “Raosoft^®^ sample size calculator” (http://www.raosoft.com/samplesize.html), on the basis of the Chinese epidemiologic data recently published (3). This reporting an overall number of 90.000 new cases of thyroid cancer for the year 2015, assuming that 90% of these tumors are represented by DTC with a 20% rate of relapse after thyroidectomy and iodine therapy.

### Statistical Analysis

All data were collected using Microsoft Excel (Microsoft Corporation, Redmond, WA, USA). Continuous variables were expressed as mean (standard deviation), and categorical variables were expressed as a percentage (frequency). Continuous variables were analyzed by the *t*-test or one-way analysis of variance (ANOVA). Categorical variables were examined using the χ^2^-test or Fischer’s exact test. Binary logistic regression calculates the dependent variable OR and 95% confidence interval (CI). The characteristics of pathological invasion were regarded as dependent variables, WHO-BMI group was used as a covariate, and age, sex, FT3, TPO-Ab, tumor diameter >1cm, multifocality, High T stage and extrathyroidal extension were used as adjustment variables. TSH, FT3, FT4, TPO-Ab and Tg-Ab do not conform to the normal distribution, so logarithmic transformation is performed. *P <*0.05 was considered statistically significant. Statistical analysis was performed using SPSS 22.0 software (IBM, Chicago, IL, USA). 

## Results

### Baseline Characteristics of Patients

Our database included 14,877 patients with PTC, of which 12,772 patients were included in the final analysis (**Figure 1**). The male-to-female ratio is 1:3.7. The average age is 42.73 ± 9.41 years. The average BMI is 24.27 ± 3.49 kg/m^2^. Obesity accounted for 6.1% of patients. In total, 166,802 LNs were dissected, of which 24,670 LNs were metastases (14.8%). The rate of metastatic LNs was 44.9% (5437/12,772). The mean number of LN metastases was 1.93 ± 3.81. The average number of LN dissected was 13.06 ± 12.31, and the LNR was 0.14 ± 0.23% as shown in **Table 1**.

**Table 1:** Basic demographics and clinical data of patients included in this study.

Characteristic	Total (N=12,772) Mean (SD) or %(n)
Sex	
Female (%)	78.9% (10,073)
Male (%)	21.1% (2,699)
Mean age (years)	42.73 (9.41)
Mean BMI (kg/m^2^)	24.27 (3.49)
BMI group ^(WHO-BMI)^	
Underweight (%)	3.2% (409)
Normal-weight (%)	58.4% (7,460)
Overweight (%)	32.3% (4,128)
Obesity (%)	6.1% (775)
Thyroid Function	
TSH (mIU/L)	3.20 (3.43)
FT3 (pmol/L)	4.53 (1.06)
FT4 (pmol/L)	15.80 (5.79)
Tg-Ab (IUmL)	
TPO-Ab (IUmL)	
Mean tumor size (mm)	0.83 (0.62)
Multifocality	40.9% (5,230)
Extrathyroidal extension	26.9% (3,441)
Rate of total metastatic LN (%)	44.9% (5,737)
Number of total LN+	1.93 (3.81)
Number of total *dissected LN*	13.06 (12.31)
Total LNR	0.14 (0.23)

BMI, body mass index; TSH, thyroid stimulating hormone; FT3, free triiodothyronine; FT4, free thyroxine; LN, lymph node; LNR, lymph node ratio.

### Influencing Factors of Metastatic LNs

Single factor logistic regression was used to analyze the factors influencing metastatic LNs. We identified other confounding factors related to the presence of metastatic LNs in addition to overweight and obesity. As shown in **Table 2**, age, FT3, TPO-Ab, tumor diameter more than 1cm, multifocality, High T stage and extrathyroidal extension are confounding factors for metastatic LNs.

**Table 2:** Regression analysis of influencing factors of metastatic LNs.

	Metastatic LNs	
	Crude OR	*P value*
Age	0.949 (0.945-0.953)	<0.001**
BMI group		
Underweight	1.529 (1.253-1.867)	<0.001**
Normal-weight	Reference	
Overweight	1.125 (1.042-1.214)	0.003**
Obesity	1.554 (1.339-1.802)	<0.001**
lgTSH	1.020 (0.925-1.125)	0.687
lgfT3	2.242 (1.445-3.478)	<0.001**
lgfT4	1.028 (0.633-1.67)	0.911
lgTPO-Ab	0.89 (0.832-0.952)	0.001**
lgTg-Ab	1.012 (0.959-1.068)	0.675
Tumor diameter>1cm	3.866 (3.538-4.225)	<0.001**
Multifocality	1.575 (1.467-1.691)	<0.001**
High T stage	1.270 (1.247-1.294)	<0.001**
Extrathyroidal extension	1.838 (1.699-1.989)	<0.001**

BMI, body mass index; TSH, thyroid stimulating hormone; FT3, free triiodothyronine; FT4, free thyroxine; OR, oods ratio; LN, lymph node.

*P < 0.05, **P < 0.01.

### Impact of BMI on the Rate of Metastatic LNs

As shown in **Table 3**, the rate of metastatic LNs in overweight and obesity patients with PTCs was significantly higher than that of normal-weight patients (45.9 vs. 52.6 vs. 42.0%, *P <*0.001). The rates of central metastatic LNs and lateral neck metastatic LNs were significantly higher in overweight and obesity patients than normal-weight patients (41.5% vs. 47.6 vs. 38.0%, *P <*0.001; 18.8 vs. 22.2 vs. 16.9%, *P <*0.001). The rates of lateral neck metastatic LNs on both the left and right sides were higher than normal-weight patients (10.2 vs. 11.1 vs. 8.6%, *P* =0.008; 10.5 vs. 13.7 vs. 9.8%, *P <*0.001).

**Table 3:** Relationship between obesity and different regional patterns of lymph node involvement.

WHO-BMI		Normal %(n)/Mean (SD)	Overweight %(n)/Mean (SD)	Obesity %(n)/Mean (SD)	*P value*
Rate of total LN +		42.0% (3133)	45.9% (1894)	52.6% (408)	<0.001**
Rate of CLN +		38.0% (2835)	41.5% (1713)	47.6% (369)	<0.001**
Rate of LLN +	Total	16.9% (1261)	18.8% (775)	22.2% (172)	<0.001**
	Right	9.8% (731)	10.5% (435)	13.7% (106)	<0.001**
	Left	8.6% (642)	10.2% (421)	11.1% (86)	0.008**
Number of total LN+		1.79 (3.67)	1.94 (3.83)	2.41 (4.20)	<0.001**
Number of CLN+		1.17 (2.28)	1.28 (2.39)	1.59 (2.72)	<0.001**
Number of LLN+	Total	0.62 (1.92)	0.66 (1.96)	0.83 (2.08)	0.003**
	Right	0.35 (1.38)	0.33 (1.26)	0.43 (1.39)	0.032
	Left	0.27 (1.17)	0.33 (1.32)	0.40 (1.43)	0.006*
Number of total dissected LN		12.1 (11.69)	13.70 (13.38)	14.37 (13.21)	<0.001**
Number of dissected CLN		5.74 (4.34)	5.71 (4.27)	5.86 (4.52)	0.028*
Number of dissected LLN	Total	6.36 (10.13)	7.99 (11.99)	8.51 (11.72)	<0.001**
	Right	3.22 (6.94)	3.87 (7.69)	4.22 (7.97)	<0.001**
	Left	3.14 (7.06)	4.12 (8.38)	4.30 (8.33)	<0.001**
Total LNR		0.14 (0.23)	0.14 (0.22)	0.18 (0.27)	<0.001**
Central LNR		0.58 (1.14)	0.21 (0.31)	0.79 (1.36)	<0.001**
Lateral LNR	Total	0.03 (0.10)	0.08 (0.12)	0.04 (0.10)	<0.001**
	Right	0.02 (0.09)	0.08 (0.13)	0.03 (0.09)	<0.001**
	Left	0.02 (0.07)	0.07 (0.12)	0.02 (0.07)	<0.001**

LN +, lymph node metastasis; CLN +, central lymph node metastasis; LLN +, lateral lymph node metastasis.

*P < 0.05, **P < 0.01.

Binary logistic regression analyzed the relationship between BMI and the risk of metastatic LNs. It was found that overweight and obesity were independent risk factor for metastatic LNs (OR_1_ = 1.125, 95% CI 1.042-1.214, *P_1 =_* 0.003; OR_2_ = 1.554, 95% CI 1.339-1.802, *P_2_* <0.001) (**Table 4**). According to the central and lateral neck lymph nodes, the adjusted OR value of metastatic LNs was calculated respectively. It was found that overweight and obesity were independent risk factor for metastatic CLNs (OR _adjusted 1_ = 1.129, 95% CI 1.038-1.227, *P_1_ = *0.005; OR _adjusted 2_ = 1.174, 95% CI 1.001-1.378, *P_2_ = *0.049). After adjusting for confounding factors, overweight and obesity were not independent risk factors for metastatic lateral LNs (*P_1_ = *0.621, *P_2_ = *0.657) (**Table 4**).

**Table 4:** Binary logistic regression analyzed the relationship between BMI and the risk of metastatic LNs.

WHO-BMI		Underweight	Normal-weight	Overweight	Obesity
Risk of total metastatic LNs	Crude OR	1.529 (1.253-1.867)	Reference	1.125 (1.042-1.214)	1.554 (1.339-1.802)
	*P value*	<0.001**		0.003**	<0.001**
	Adjusted OR	1.139 (0.915-1.417)	Reference	1.119 (1.03-1.216)	1.198 (1.02-1.408)
	*P value*	0.244		0.008**	0.028*
Risk of metastatic CLNs	Crude OR	1.602 (1.312-1.955)	Reference	1.120 (1.036-1.21)	1.511 (1.303-1.752)
	*P value*	<0.001**		0.004**	<0.001**
	Adjusted OR	1.164 (0.937-1.447)	Reference	1.129 (1.038-1.227)	1.174 (1.001-1.378)
	*P value*	0.171		0.005**	0.049*
Risk of metastatic LLNs	Crude OR	1.487 (1.175-1.882)	Reference	1.106 (1.002-1.22)	1.436 (1.203-1.715)
	*P value*	0.001**		0.046*	<0.001**
	Adjusted OR	1.17 (0.9-1.521)	Reference	1.028 (0.923-1.144)	1.045 (0.86-1.271)
	*P value*	0.242		0.621	0.657

BMI, body mass index; OR, odds ratio; CI, confidence interval; LN +, lymph node metastasis; CLN, central lymph node; LLN, lateral lymph node.

Age, lgFT3, lgTPO-Ab, tumor diameter >1cm, multifocality, High T stage and extrathyroidal extension as covariates to adjust OR value.

*P < 0.05, **P < 0.01.

### Impact of BMI on the Number of Metastatic LNs

As shown in **Table 3**, the total number of LN metastases was significantly higher in obese patients than normal-weight patients (2.41 vs. 1.79, *P <*0.001). Among the obese patients, the number of LN metastases in the central and lateral neck regions were higher than the normal-weight patients (1.59 vs. 1.17, *P <*0.001; 0.83 vs. 0.62, *P* =0.006). In addition, the number of lateral neck LN metastases was higher than that of the normal-weight patients on the left side (0.40 vs. 0.27, *P* =0.018).

It was found that overweight and obesity were independent risk factor for metastatic LNs more than three (OR _adjusted 1_ = 1.108, 95% CI 1.011-1.214, *P_1_ = *0.029; OR _adjusted 2_ = 1.6225% CI 1.377-1.910 *P_2_* <0.001) (**Table 5**). It was found that overweight and obesity were independent risk factor for number of metastatic CLNs more than two (OR_1_ = 1.106, 95% CI 1.006-1.215, *P_1_ = *0.038; OR_2_ = 1.159, 95% CI 0.975-1.377, *P_2_ = *0.045). After adjusting for confounding factors, overweight and obesity were not independent risk factors for number of metastatic lateral LNs more than two (*P_1_ = *0.866, *P_2_ = *0.907) (**Table 5**). 

**Table 5:** Binary logistic regression analyzed the relationship between BMI and the number of metastatic LNs.

WHO-BMI		Underweight	Normal-weight	Overweight	Obesity
Number^1^ of total metastatic LNs	Crude OR	1.859 (1.502-2.301)	Reference	1.108 (1.011-1.214)	1.622 (1.377-1.910)
	*P value*	<0.001**		0.029*	<0.001**
	Adjusted OR	1.294 (1.017-1.648)	Reference	1.091 (0.987-1.207)	1.201 (1.002-1.439)
	*P value*	0.036*		0.090	0.047*
Number^2^ of metastatic CLNs	Crude OR	1.718 (1.394-2.117)	Reference	1.091 (1.000-1.190)	1.526 (1.302-1.789)
	*P value*	<0.001**		0.051	<0.001**
	Adjusted OR	1.148 (0.91-1.448)	Reference	1.106 (1.006-1.215)	1.159 (0.975-1.377)
	*P value*	0.243		0.038*	0.045*
Number^3^ of metastatic LLNs	Crude OR	1.665 (1.287-2.154)	Reference	1.076 (0.96-1.206)	1.447 (1.184-1.769)
	*P value*	<0.001**		0.208	<0.001**
	Adjusted OR	1.263 (0.947-1.686)	Reference	0.989 (0.874-1.12)	1.013 (0.813-1.262)
	*P value*	0.112		0.866	0.907

BMI, body mass index; OR, odds ratio; CI, confidence interval; LNs, lymph nodes; CLNs, central lymph nodes; LLNs, lateral lymph nodes.

Age, tumor diameter >1cm, multifocality, High T stage and extrathyroidal extension as covariates to adjust OR value.

^1^The cut-off point of number in total LN + is three.

^2^The cut-off point of number in total CLN + is two.

^3^The cut-off point of number in total LLN + is two.

*P < 0.05, **P < 0.01.

### Impact of BMI on the Number of Dissected LNs

The total number of *dissected LN* in the obese patients with PTC was significantly higher than the normal-weight patients (14.37 vs. 12.10, *P <*0.001). The obese patients have a higher total number of lateral neck *dissected LN* (8.51 vs. 6.36, *P <*0.001). This difference exists on the left and right sides of the neck (**Table 4**). However, there was no difference in the number of CNDs (*P* =0.466).

Binary logistic regression analyzed the relationship between BMI and number of dissected LNs. Overweight and obesity were independent risk factor for the number of dissected LNs more than twelve (OR_1_ = 1.156, 95% CI 1.069-1.250, *P_1_* <0.001; OR_2_ = 1.376, 95% CI 1.184-1.59, *P_2_* <0.001) (**Table 6**). It was found that obesity was not an independent risk factor for number of dissected CLNs more than five (*P*=0.653). After adjusting for confounding factors, overweight and obesity were independent risk factors for number of dissected lateral LNs more than six (OR_1_ = 1.122, 95% CI 1.032-1.219, *P_1_ = *0.007; OR_2_ = 1.185, 95% CI 1.010-1.391, *P_2_ = *0.037) (**Table 6**).

**Table 6:** Binary logistic regression analyzed the relationship between BMI and the number of LNs dissections.

WHO-BMI		Underweight	Normal-weight	Overweight	Obesity
Number of total LNs dissections^1^	Crude OR	1.317 (1.076-1.611)	Reference	1.156 (1.069-1.250)	1.376 (1.184-1.598)
	*P value*	0.007**		<0.001**	<0.001**
	Adjusted OR	0.992 (0.808-1.218)	Reference	1.128 (1.038-1.226)	1.173 (1-1.377)
	*P value*	0.942		0.004*	0.047*
Number of CLNs dissections^2^	Crude OR	1.16 (0.95-1.416)	Reference	0.875 (0.81-0.945)	1.127 (0.971-1.307)
	*P value*	0.145		0.001**	0.115
	Adjusted OR	0.964 (0.782-1.189)	Reference	0.895 (0.826-0.97)	1.036 (0.888-1.208)
	*P value*	0.735		0.007**	0.653
Number of LLNs dissections^3^	Crude OR	1.26 (1.029-1.542)	Reference	1.168 (1.08-1.264)	1.384 (1.191-1.607)
	*P value*	0.025*		<0.001**	<0.001**
	Adjusted OR	1.14 (0.918-1.414)	Reference	1.122 (1.032-1.219)	1.185 (1.010-1.391)
	*P value*	0.235		0.007**	0.037*

BMI, body mass index; OR, odds ratio; LN, lymph node; CLN, central lymph node; LLN, lateral lymph node.

Age, lgFT3, lgTPO-Ab, tumor diameter >1cm, multifocality, High T stage and extrathyroidal extension as covariates to adjust OR value.

^1^The cut-off point of number in total LNs dissections is twelve.

^2^The cut-off point of number in total CLNs dissections is five.

^3^The cut-off point of number in total LLNs dissections is six.

*P < 0.05, **P < 0.01.

### Impact of BMI on the LNR

The LNR in obese patients was significantly higher than normal-weight patients (0.18 vs. 0.14, *P <*0.001) (**Table 5**). While the difference was statistically significant in the central LNs (0.79 vs. 0.58, *P <*0.001), but there was no statistically significant difference in the lateral neck LNs (*P* =0.067) (**Table 5**).

It was found that overweight and obesity were independent risk factor for the LNR more than 0.07 (OR _adjusted 1_ = 1.086, 95% CI 1.005-1.174, *P_1_ = *0.036; OR _adjusted 2_ = 1.537, 95% CI 1.325-1.783 *P_2_* <0.001) (**Table 7**). It was found that overweight and obesity were independent risk factor for the central LNR more than 0.12 (OR_1 =_ 1.099, 95% CI 1.011-1.194, *P_1_ = *0.027; OR_2_ = 1.177, 95% CI 1.003-1.381, *P_2_ = *0.045). But obesity was not an independent risk factor for the lateral LNR more than 0.05 (*P_2_ = *0.283) (**Table 7**).

**Table 7:** Binary logistic regression analyzed the relationship between BMI and the risk of LNR.

WHO-BMI		Underweight	Normal-weight	Overweight	Obesity
Total LNR^1^	Crude OR	1.637 (1.341-1.998)	Reference	1.086 (1.005-1.174)	1.537 (1.325-1.783)
	*P value*	<0.001**		0.036*	<0.001**
	Adjusted OR	1.216 (0.978-1.511)	Reference	1.081 (0.994-1.176)	1.195 (1.018-1.403)
	*P value*	0.078		0.067	0.029*
Central LNR^2^	Crude OR	1.526 (1.25-1.862)	Reference	1.095 (1.014-1.184)	1.511 (1.303-1.752)
	*P value*	<0.001**		0.021*	<0.001**
	Adjusted OR	1.106 (0.89-1.374)	Reference	1.099 (1.011-1.194)	1.177 (1.003-1.381)
	*P value*	0.364		0.027*	0.045*
Lateral LNR^3^	Crude OR	19.11 (15.047-24.269)	Reference	15.914 (14.485-17.483)	1.357 (1.123-1.641)
	*P value*	<0.001**		<0.001**	0.002**
	Adjusted OR	19.425 (15.183-24.854)	Reference	18.239 (16.501-20.16)	1.113 (0.915-1.354)
	*P value*	<0.001**		<0.001**	0.283

OR, odds ratio; LNR, lymph node ratio.

Age, lgFT3, lgTPO-Ab, tumor diameter >1cm, multifocality, High T stage and extrathyroidal extension as covariates to adjust OR value.

^1^The cut-off point of total LNR is 0.07.

^2^The cut-off point of central LNR is 0.12.

^3^The cut-off point of lateral LNR is 0.05.

*P < 0.05, **P < 0.01.

### Impact of BMI on the Size of Positive LNs

**Figure 2:**
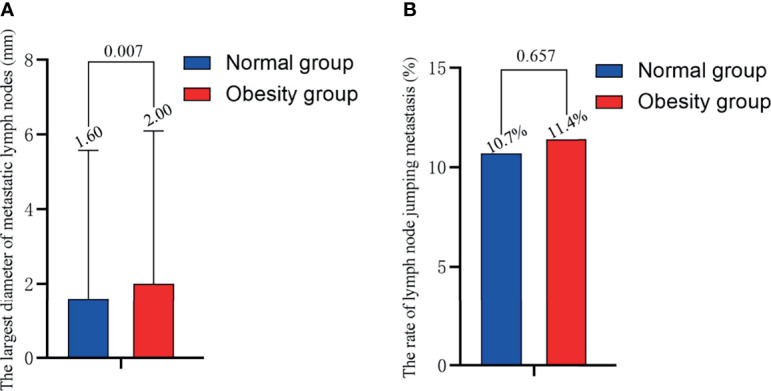
Relationship of obesity with **(A)** the maximum diameter of positive LN and **(B)** the rate of LN skip metastasis. BMI, body mass index; LN, lymph node.

As shown in **Figure 2A**, the maximum diameter of positive LNs in obese patients was higher than normal-weight patients (2.00 vs. 1.60 mm, *P* =0.007). There was no significant difference in the rate of LN skip metastasis between the two groups (**Figure 2B**). 

## Discussion

**Figure 3:**
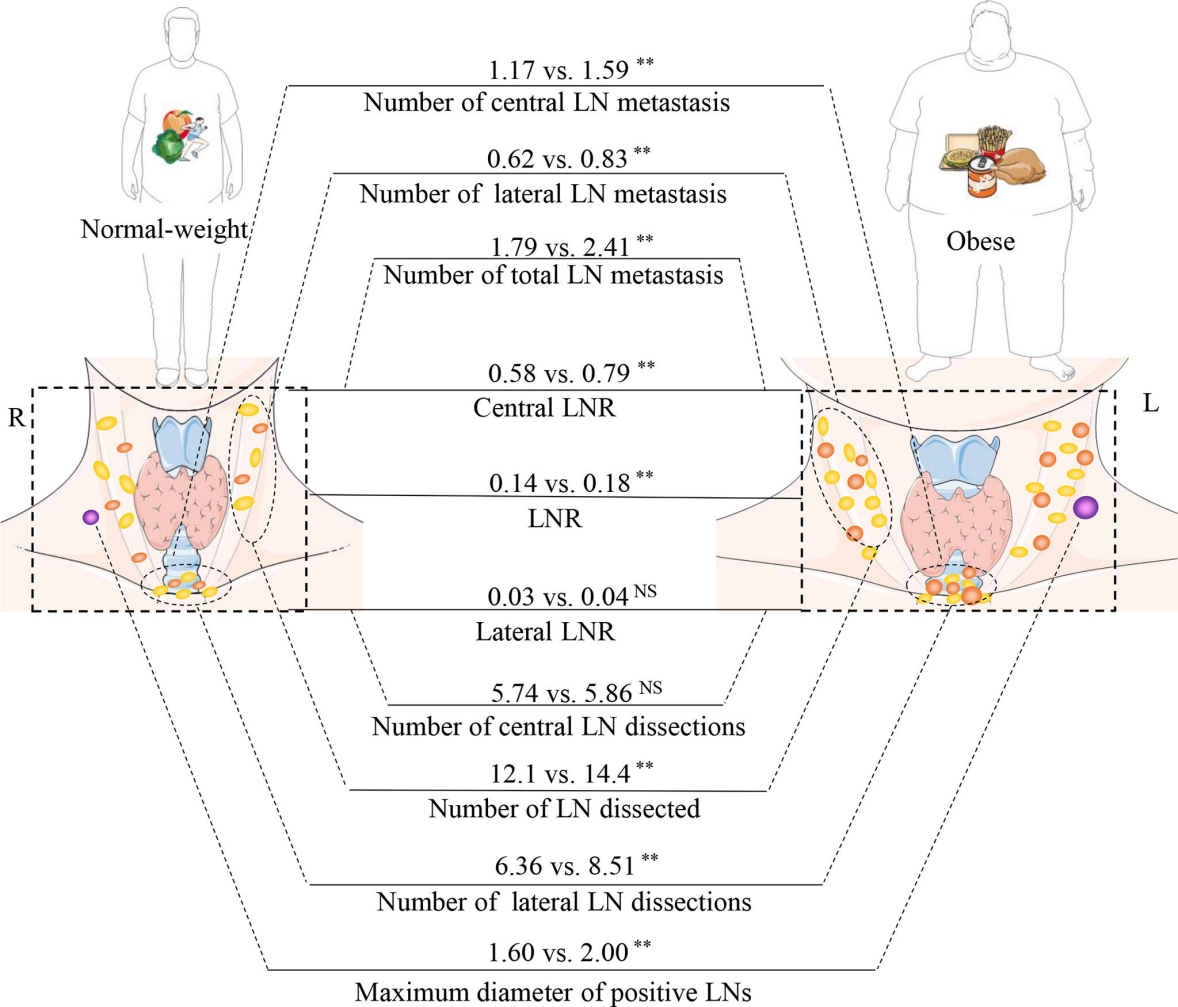
Relationship between obesity and the rules of lymph node (LN) metastasis. The part of schematic art pieces used in this figure were provided by Servier Medical art (http://servier.com/Powerpoint-image-bank). Servier Medical Art by Servier is licensed under a Creative Commons Attribution (CC BY) 3.0 Unported License. BMI, body mass index; LNR, Lymph node ratio; LN, lymph node. **P < 0.01; ns, not statistically significant.

This study is a retrospective analysis of 12,772 patients with PTC, with a focus on the correlation between LN status and obesity. Obesity not only increased the rate of metastatic LNs, but also increased the number of metastatic LNs and the maximum diameter of positive LNs, which are two indicators of a poor prognosis. We analyzed two indicators that have been overlooked in previous studies, including the number of *dissected LN* and the LNR. These results showed opposite regional differences between obesity, the number of *dissected LN*, and the LNR (**Figure 3**).

### Effect on the Rate of Metastatic LNs

Previous studies on the relationship between obesity and metastatic LNs in thyroid cancer have been controversial (**Table 8**) (18–28). Yu et al. found a positive correlation between BMI and neck LN metastasis (OR = 1.58, *P* =0.02) (24). Our previous research also yielded consistent results with those of Yu et al. (OR = 1.493) (8, 23, 24). One of the possible mechanisms is that serum leptin levels are higher in obese patients with PTC. *In vitro* studies have indicated that leptin promotes invasion and migration of thyroid cancer cell lines (29, 30). However, Kim, Gasior, Grani, Tresallet, and others have found that obesity is not associated with neck metastatic LNs in patients with thyroid cancer (19, 20, 26, 28). Simultaneously, Paes et al. found that obesity is negatively correlated with metastatic LNs (18). This finding may be due to differences in ethnicity. In the current study, we found that the rate of metastatic LNs in obese patients with PTC was significantly higher than normal-weight patients (52.6% vs. 42.0, *P <*0.001). After adjusting for confounding factors, it was found that overweight and obesity were independent risk factor for metastatic LNs (OR_1_ = 1.125, 95% CI 1.042-1.214, *P_1_ = *0.003; OR_2_ = 1.554, 95% CI 1.339-1.802, *P_2_* <0.001). This effect exists in both the central and lateral neck regions (**Figure 3**).

**Table 8:** Previous studies on the relationship between obesity and LN metastasis of thyroid cancer.

References	Date	Race	Cases (N)	BMI (kg/m^2^)	Rate of obesity (%)	Correlation between BMI and LN metastasis
Paes et al. (18)	2010	Mostly Caucasian (93%)	259	27.8	38.9%^1^	Negative correlation
Kim et al. (19)	2013	Asian	2057	23.8	5%^1^	Non-correlation
Tresallet et al. (20)	2014	Caucasian	1216	N/A	14.5%^1^	Non-correlation
Lee et al. (21)	2015	Asian	1121	23.3	27%^1^	Non-correlation
Choi et al. (22)	2015	Asian	612	23.1	2.1%^1^	Non-correlation
Kim et al. (23)	2016	Asian	5081	N/A	5%^1^	Correlation
Yu et al. (24)	2017	Asian	1622	N/A	24.3%^2^	Correlation
Wu et al. (25)	2017	Asian	796	25	8%^1^	Correlation
Gąsior et al. (26)	2018	Caucasian	1181	28.1	33.7%^1^	Non-correlation
Feng et al. (27)	2013	Asian	417	23.9	6%^1^	Correlation
Grani et al. (28)	2018	Caucasian	432	N/A	19.8%^1^	Non-correlation

^1^Defined the standard of obesity as BMI ≥27.5 kg/m^2^.

^2^Defined the standard of obesity as BMI ≥30.0 kg/m^2^.

N/A, Not available.

### Number of Metastatic LNs and the Sizes of Positive LNs

The 2015 ATA guidelines suggest that more than five metastatic LNs yields an intermediate risk of recurrence (11). Previously, Leboulleux et al. found a recurrence rate of 3% with less than five metastases, while 6 to 10 metastases was associated with a recurrence rate of 7% and >10 metastases with 21% (31). Previous studies have given minimal attention to the relationship between the number of metastatic LNs and obesity. The current study revealed a positive correlation between obesity and metastatic LNs (2.41 vs. 1.79, *P <*0.001), which was reflected in the central and lateral neck LNs (**Figure 3**).

The maximum diameter of positive LNs is another indicator of poor prognoses. The ATA guidelines classify the maximum diameter of positive LN between 0.2 and 3.0 cm as the intermediate risk of recurrence (11). In a previous study, the rate of locoregional recurrence was 5% for patients with maximum positive LN diameters smaller than 0.2 cm (12). When the maximum diameter of positive LNs were >3 cm, the rate of locoregional recurrence increased to 27% (13). In the current study, obese patients with PTC had larger maximum positive LN diameters (2.00 vs. 1.60, *P* =0.007), suggesting that obesity may lead to poorer prognoses. Previous studies have primarily shown that obesity increases the rate of LN metastasis. This paper confirmed that obesity not only increases the rate of metastatic LNs, but also increases the number of metastatic LNs and the maximum diameter of positive LNs. 

### Number of Dissected LN

Neck LNs are commonly wrapped in adipose tissue, and obese patients have more adipose tissue. Only a few studies have focused on whether the increased adipose tissue in obese patients can affect the dissection of neck LNs. This study found that obese patients had more dissectible LNs, yet this association only exists in the lateral neck area (14.37 vs. 12.10, *P <*0.001), which seems to be more beneficial for obese patients. However, the number of CNDs is not related to obesity. There is a regional difference between the number of *dissected LN* and obesity (**Figure 3**). One of the possible reasons is that the difficulty of intraoperative identification of LNs is reduced. LNs are often surrounded by adipose tissue, which helps the surgeon identify the LNs and minimize the burden of identification. The second reason involves inflammatory factor stimulation, as obesity can cause adipose cells to secrete inflammatory factors, such as C-reactive protein, interleukin 6 (IL-6), IL-10, and tumor necrosis factor (TNF-α). These inflammatory factors may stimulate LN hyperplasia (32). 

### LNR

After discovering a positive relationship between obesity and the number of lateral neck *dissected LN*, we speculated whether more *dissected LN* would yield higher positivity rates, which could contribute to the radical cure of thyroid cancer in obese patients. However, by analyzing the LNR, we found that obesity did not increase the positive rate of lateral central LNs. On the other hand, obesity was found to increase the positive rate of central LNs. Hence, there are regional differences in the LNR and the number of *dissected LN*.

Obese patients have difficulty in performing CNDs due to their short and thick necks. In this study, obesity was not associated with an increase in the number of CNDs, but it was associated with an increased LNR. Hence, surgeons should be cautious when performing CNDs for obese patients, as there may be more positive LNs in obese patients. Obesity may increase risk of inadequate lymph node dissection during surgery.

In the current study, we revealed that obesity promotes the metastasis of LNs and increases the average diameter of positive LNs. These two indicators are indicative of a poor prognosis. In addition, we considered the number of *dissected LN* and the LNR into the current analysis. We found a regional difference between obesity and these two indicators. 

### Limitations

Most sources of error due to confounding and bias are more common in retrospective studies than in prospective studies. However, this retrospective study will be helpful to assess the feasibility of future prospective studies and to help in their design. This paper did not analyze the relationship between obesity and LNs in various regions of the lateral neck. The number of positive nodes is often affected by the variability in nodal staging techniques, which may yield different numbers of excised nodes. Finally, this study did not analyze the disease-free survival rate or locoregional recurrence rate of patients, for which LN metastasis is a very important independent risk factor in patients with thyroid cancer after curative resection.

This is a large-scale study of more than 10.000 PTC patients in which several of significance tests were performed. The testing procedure can be biased by latent confounding factors such as batch effects and unmeasured covariates that correlate with both primary variables of interest and the outcome. Despite the methodological advances in this paper, providing analysis that are able to capture complex and regional shape differences, the limits of the analysis methodology may remain. It is clear, for example, that a limit and bias of this work is BMI remodelling of PTC patients due to the practice (or not practice) of exercise, which it is perhaps impossible to analyze with the present data available. In addition, these data represent the work of multiple surgeons with varying degree of expertise.

## Data Availability Statement

The raw data supporting the conclusions of this article will be made available by the authors, without undue reservation. Requests to access the datasets should be directed to Hui Sun, s_h@jlu.edu.cn.

## Ethics Statement

This study was approved by the Health Care Ethics Committee of the China-Japan Union Hospital of Jilin University (No. 2019040806).

## Author Contributions

Conception and design, HS and HG. Administrative support, HS. Collection and assembly of data, CL and NL. Data analysis and interpretation, NL. Manuscript writing, CL and GD. All authors contributed to the article and approved the submitted version

## Funding

This study was supported by the National Nature Science Foundation of China NSFC, Grant No: 81972499. 

## Conflict of Interest

The authors declare that the research was conducted in the absence of any commercial or financial relationships that could be construed as a potential conflict of interest.

## Publisher’s Note

All claims expressed in this article are solely those of the authors and do not necessarily represent those of their affiliated organizations, or those of the publisher, the editors and the reviewers. Any product that may be evaluated in this article, or claim that may be made by its manufacturer, is not guaranteed or endorsed by the publisher.
